# Resequencing worldwide spinach germplasm for identification of field resistance QTLs to downy mildew and assessment of genomic selection methods

**DOI:** 10.1093/hr/uhac205

**Published:** 2022-09-13

**Authors:** Gehendra Bhattarai, Ainong Shi, Beiquan Mou, James C Correll

**Affiliations:** Department of Horticulture, University of Arkansas, Fayetteville, AR 72701, USA; Department of Horticulture, University of Arkansas, Fayetteville, AR 72701, USA; USDA-ARS, Crop Improvement and Protection Research Unit, Salinas, CA, 93905, USA; Department of Entomology and Plant Pathology, University of Arkansas, Fayetteville, AR 72701, USA

## Abstract

Downy mildew, commercially the most important disease of spinach, is caused by the obligate oomycete *Peronospora effusa*. In the past two decades, new pathogen races have repeatedly overcome the resistance used in newly released cultivars, urging the need for more durable resistance. Commercial spinach cultivars are bred with major R genes to impart resistance to downy mildew pathogens and are effective against some pathogen races/isolates. This work aimed to evaluate the worldwide USDA spinach germplasm collections and commercial cultivars for resistance to downy mildew pathogen in the field condition under natural inoculum pressure and conduct genome wide association analysis (GWAS) to identify resistance-associated genomic regions (alleles). Another objective was to evaluate the prediction accuracy (PA) using several genomic prediction (GP) methods to assess the potential implementation of genomic selection (GS) to improve spinach breeding for resistance to downy mildew pathogen. More than four hundred diverse spinach genotypes comprising USDA germplasm accessions and commercial cultivars were evaluated for resistance to downy mildew pathogen between 2017–2019 in Salinas Valley, California and Yuma, Arizona. GWAS was performed using single nucleotide polymorphism (SNP) markers identified via whole genome resequencing (WGR) in GAPIT and TASSEL programs; detected 14, 12, 5, and 10 significantly associated SNP markers with the resistance from four tested environments, respectively; and the QTL alleles were detected at the previously reported region of chromosome 3 in three of the four experiments. In parallel, PA was assessed using six GP models and seven unique marker datasets for field resistance to downy mildew pathogen across four tested environments. The results suggest the suitability of GS to improve field resistance to downy mildew pathogen. The QTL, SNP markers, and PA estimates provide new information in spinach breeding to select resistant plants and breeding lines through marker-assisted selection (MAS) and GS, eventually helping to accumulate beneficial alleles for durable disease resistance.

## Introduction

Spinach (*Spinacia oleracea* L.) is an important cool-season leafy vegetable crop. The US is the second-largest producer of spinach after China. The demand for fresh market spinach has doubled in the last decade, with current production of 0.44 million tonnes [1]. More than 90% of US spinach is produced from March through October in Salinas Valley, CA, and warmer regions from November to March in Yuma, AZ, providing a yearly supply of fresh produce. Organic production comprises around half of the total spinach production in the US.

Downy mildew (DM), the most important disease of spinach, is caused by an obligate oomycete *Peronospora effusa*, which often causes a complete crop loss as infected leaves are not marketable. A total of 19 unique races and many isolates of *P. effusa* have been reported [2–5]. In the past two decades, regular outbreaks of new races are repeatedly overcoming the race-specific R genes in spinach cultivars. Sets of R loci (*RPF*) are hypothesized to control resistance to downy mildew pathogen in spinach [6]. Spinach commercial cultivars are hybrids containing a combination of the major RPF loci from male and female parents that are effective against different races of *P. effusa*, a traditional practice of gene pyramiding. Past efforts mapped the RPF1, RFP2, and RPF3 locus to chromosome 3 [7, 8]. Based on the SpoV1 genome assembly annotation, five disease resistance genes adjacent to the RPF loci were reported as potential downy mildewresistance candidate genes [9]. The RPF1 locus was narrowed to 0.89 Mb [10] and between 0.39 to 1.23 Mb region of chromosome 3 Spov1 assembly [11]. Another study mapped the RPF3 resistance region in cultivar Whale to 0.57 Mb of Spov1 Chromosome 3, spanning between 0.66–1.23 Mb [12]. These recent investigations reported the most likely candidate genes involved in providing disease resistance based on the predicted functions of the protein coding sequence in the genome assembly [10–12]. These results present the progress in developing markers associated or linked to resistance to downy mildew pathogen and available molecular resources to implement marker assisted selection (MAS) in spinach. The new spinach genome assembly of inbred line Monoe-Viroflay was recently generated and several important spinach traits were mapped including resistance to downy mildew pathogen in 0.25–1.55 Mb region of chromosome 3 containing six genes encoding NBS-LRR proteins and five encoding receptor kinases, known to provide disease resistance in plants.

Plant pathogens continuously evolve and overcome the race-specific resistance conferred by major genes, as described in resistance to leaf rust in wheat [13] and downy mildew in grape [14]. The widespread use of a dominant resistant gene increases directional selection pressure and a high mutation rate of pathogen favors evolution from avirulent to virulent pathotypes, as reviewed by McDonald and Linde (2002) [15]. On the other hand, QTLs, governed by alleles at multiple genomic regions, are more durable as multiple mutations, which are less probable, are needed to overcome the polygenic resistance [16]. The durability of polygenic resistance was experimentally demonstrated in pepper using potato virus Y (PVY) isolates, where the resistance governed by a major gene (*pvr2^3^*) broke down at a higher frequency than when the same gene (*pvr2^3^*) was introgressed in combination with a partial resistance QTL [17]. Reduced multiplication potential of PVY, multiple mutations required for the virus to become virulent, and slow selection of the virulent PVY pathotypes were reported as the mechanism for the durability of (*pvr2^3^*) genes in the presence of QTL against PVY [18]. Genetic resistance differs between qualitative and quantitative resistance; the former is more straightforward to introgress and eased by the MAS approach but is vulnerable to resistance breakdown, while the latter is more complex from breeding perspectives. Genomic selection (GS) will better fit the breeding program when the resistance breeding effort prioritizes many minor genes instead of or in combination with major R genes [19]. The regular emergence of new races of *P. effusa* breaking down the known RPF genes highlights the need to identify and utilize quantitative resistance to achieve durable resistance for sustainable spinach production. Quantitative resistance does not entirely impede disease development but reduces the severity and is often effective against multiple pathogen races. Using minor resistance alleles on top of major R genes may be more effective in disease management and minimizing disease spread, which is our general hypothesis and supposition of this work.

Genome wide association studies (GWAS) are used to identify genetic variants associated with the trait of interest in natural and segregating populations, and the resolution of genetic associations depends on linkage disequilibrium (LD) between markers and the trait. GWAS has been employed to map disease resistance in many crops, including resistance to downy mildew pathogen in spinach [11, 12, 20], lettuce [21], and other crops [22–25]. Spinach, a cross-pollinating crop, shows faster LD decay due to its heterozygosity, and association analysis with a denser marker coverage is expected to map the trait at a higher resolution. GS predicts the breeding value of complex traits in the test population by assessing the effect of genome wide markers, facilitates the selection of superior genotypes without phenotyping and field tests, and accelerating breeding cycles [26–29]. In the past two decades, GS has been reported in several horticultural and agronomic crops for qualitative and quantitative traits in biparental, multiparent, and natural populations [30–35], including spinach for white rust resistance [36]. Several parametric (rrBLUP-ridge regression BLUP, Bayes A, Bayes B, Bayesian LASSO) and non-parametric (RKHS-Reproducing Kernel Hilbert Space, RF- Random Forest) models are optimized to increase prediction accuracy in plant and animal breeding programs. These models have a different assumption for the trait inheritance pattern and the marker effects distribution, so their prediction ability varies depending on the architecture of traits and the number and effect sizes of QTLs. Some models perform better for traits controlled by a few major QTLs, while others are more suited for traits controlled by many minor alleles. Some known factors affecting the prediction accuracies are trait heritability, the number of QTLs, training and testing population size, genetic diversity within the population, relatedness among genotypes in the training and testing set, and the number of markers and LD patterns [19, 32, 37–39].

Variable inoculum pressure and the presence of multiple races are the main hindrances to large-scale field disease screening. Despite the potential challenges, a diverse set of spinach germplasm was evaluated under field conditions at two locations: Yuma, AZ, and Salinas, CA. Field evaluation does not allow to control predominance of pathogen isolates, and the disease resistance screening involves mixed pathogen populations and is expected to detect race non-specific broad-spectrum resistance. The objectives of the present study were to evaluate the worldwide spinach germplasm collection and commercial cultivars for resistance against downy mildew pathogen in the field condition under natural inoculum pressure and to perform GWAS to identify genomic regions associated with field resistance. Further, the potential performance of GS was assessed for the first time using six different GP methods and several marker subsets on resistance to downy mildew pathogen in the spinach GWAS panel to evaluate prospects of incorporating the GS to improve resistance. In this study, disease screening in the field conditions aimed to discover and uncover race non-specific resistance loci or the QTLs against the downy mildew pathogen in the real world under natural disease pressure and to identify new genomic regions providing resistance.

**Figure 1:**
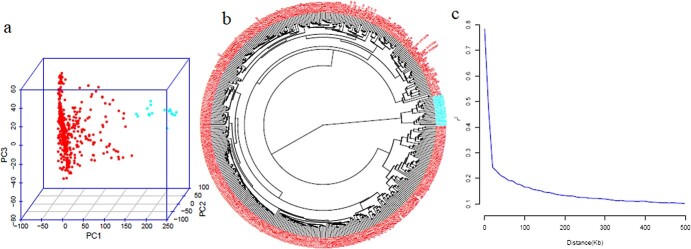
(a) Principal component analysis (PCA) of 434 spinach accessions (b) Genetic relatedness among 434 spinach accessions determined by the neighbor-joining methods (c) Genome-wide linkage disequilibrium (LD) decay pattern of spinach measured by r^2^ between SNPs pairs.

## Results

### Phenotype

**Figure 2:**
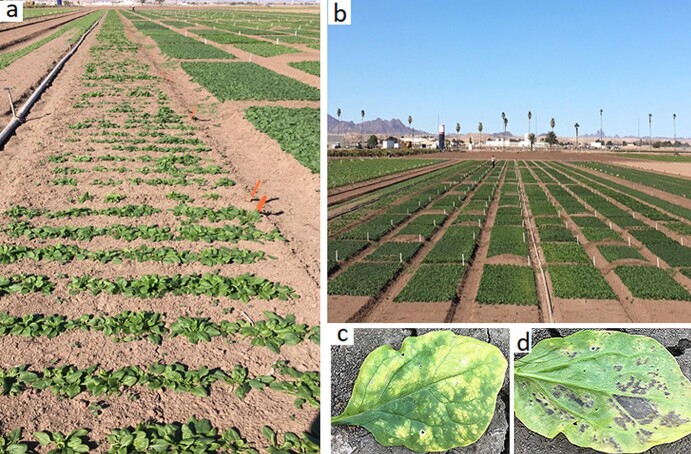
Downy mildew field trail (2a,b). Downy mildew disease symptoms in the adaxial leaf surface (2c) and signs of the pathogen in the abaxial leaf surface (2d) in the field-grown spinach.

**Figure 3:**
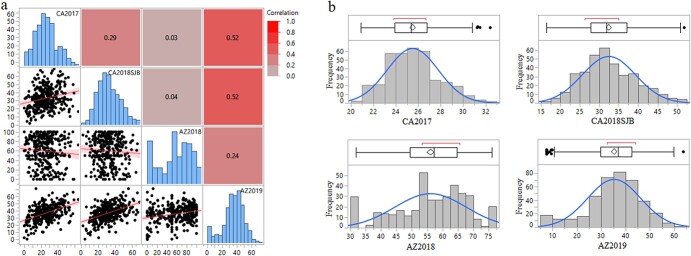
Frequency distribution of downy mildew disease response among the worldwide spinach germplasm accessions evaluated in four environments during 2017–2019. Trials were conducted in Salinas, CA in 2017 (CA2017), San Juan Bautista, CA in 2018 (CA2018SJB), and Yuma, AZ in 2018 (AZ2018) and 2019 (AZ2019).

This study screened 434 spinach genotypes collected initially from 34 countries for resistance to downy mildew pathogen under natural infestation in four different environments (Supplementary Table 1). Spinach accessions were grown in a row along with commercial cultivars and susceptible cultivar Viroflay in the border row (Fig. 2a, b). The adaxial (Fig. 2c) leaf surface shows symptoms and the abaxial (Fig. 2d) leaf surface shows both signs and symptoms of downy mildew pathogen that were scored for resistance-susceptibility as detailed previously. Downy mildew disease scores were available for 359, 363, 377, and 387 spinach accessions for CA2017, CA2018SJB, AZ2018, and AZ2019 trials. The susceptible cultivar Viroflay planted in the border row was moderate to severely infected in all evaluated environments. The predominance of races and isolates of the pathogen varied across tested environments, as reported in annual varieties trials performed in the same locations [51–53]. The environment effect and genotype x environment effect were significant (*P* < 0.01) as observed in the scatter plot of disease response between environments, even though the Pearson correlation of mean disease response across environments ranged between 0.03–0.52, showing low to moderate correlations between pairs of field trials (Fig. 3a). The spinach accessions are genetically heterogeneous, showing segregation and variation within accessions. Thus, disease responses from each location were analyzed separately to account for environmental variation, particularly considering variable pathogen population dynamics. The genotype effect was significant (*P* < 0.01) on ANOVA for downy mildew disease response in all four environments (field trials). Phenotypic analysis was performed assuming normal distribution to independently estimate the BLUP values for each environment and was used as in GWAS and GS. The BLUP histograms of the disease responses across the experimental locations (Fig. 3b) show substantial genetic variations among the genotypes in each trial. Disease severity across the environment ranged from 7.6 to 76.0, with the mean disease severity of 35.3, 56.1, 25.5, and 32.4 for AZ2019, AZ2018, CA2017, and CA2018SJB, respectively (Table 1, Supplementary Table 1). The broad-sense heritability calculated on a genotype-mean basis showed moderate to high heritability across locations ranging from 0.18 to 0.78 with a mean of 0.45 (Table 1).

**Table 1:** Phenotypic distribution and broad-sense heritability of downy mildew field resistances across four environments

**Environment**	**Heritability**	**Mean**	**Min**	**Max**	**SD**
CA2017	0.18	25.5	20.9	32.4	2.28
CA2018SJB	0.53	32.4	16.4	51.8	6.85
AZ2018	0.29	56.1	31.9	76.0	10.90
AZ2019	0.78	35.3	7.6	63.8	11.61

**Figure 4:**
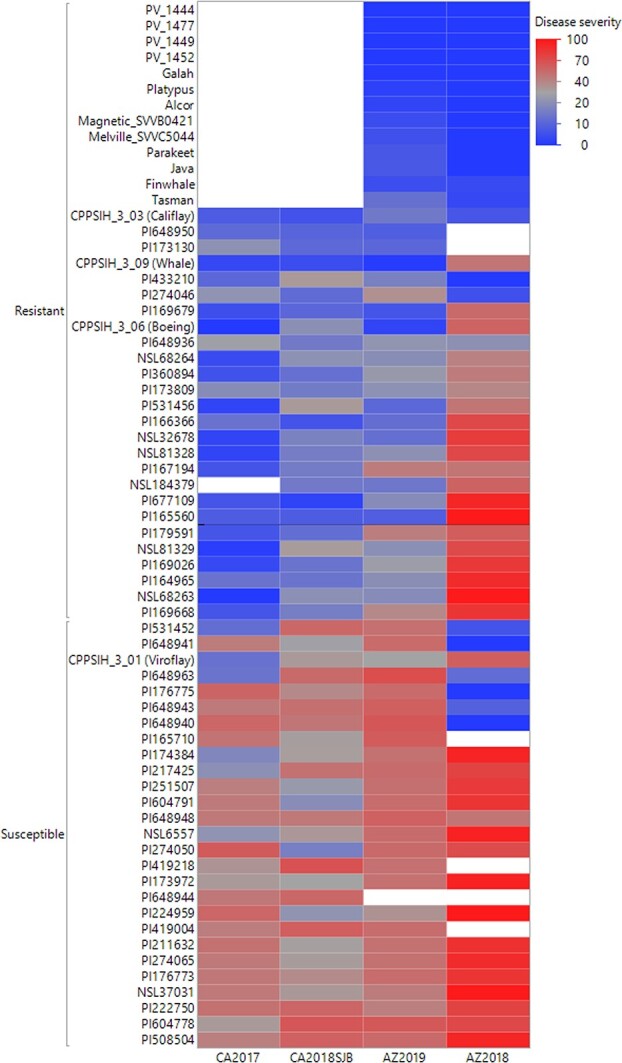
Heatmap visualization of downy mildew disease response from each environment. The environment is noted on the x-axis and the spinach accessions are listed on the y-axis. The color code on the y-axis shows the disease severity scale.

The top 10% of resistant and susceptible accessions from each environment showing stability across environments are shown in Fig. 4. Still, these selected genotypes were not completely stable and show genotype x environment interactions, as evident in the heat map (Fig. 4). However, the disease scores of these tolerant accessions were lower than the mean disease scores in individual locations (Supplementary Table 1). The accessions, PI 433210, PI 648936, PI 274046, CPPSIH_3_03 (Califlay), PI 173809, PI 173130, PI 360894, NSL 68264, CPPSIH_3_06 (Boeing), NSL 184379, PI 648950, PI 169679, CPPSIH_3_09 (Whale), NSL 68263, NSL 32678, PI 169026, NSL 81328, PI 166366, PI164965, PI 165560, PI 677109, PI 169668, PI 179591, PI 167194, PI 531456, and NSL 81329 showed high tolerance to downy mildew pathogen in more than two tested environments (Fig 4). In addition, the commercial cultivars Alcor, Galah, Java, Magnetic, Parakeet, Platypus, PV_1444, PV_1449, PV_1452, _PV_1477, Finwhale, and Tasman showed high resistance in both AZ2018 and AZ2019 environments. These consistently tolerant accessions are potentially useful as parents in breeding programs.

### Genetic diversity

**Figure 5:**
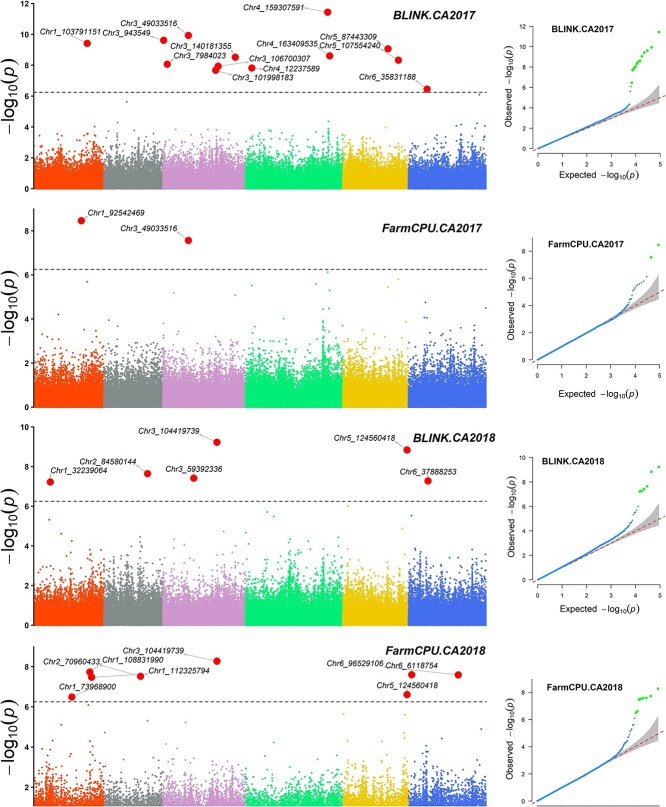
GWAS of resistance to downy mildew evaluated in the field condition in Salinas, California, in 2017 (CA2017) and San Juan Bautista in 2018 (CA2018SJB). The Manhattan and QQ plots are presented for each of the four environment trials.

**Figure 6:**
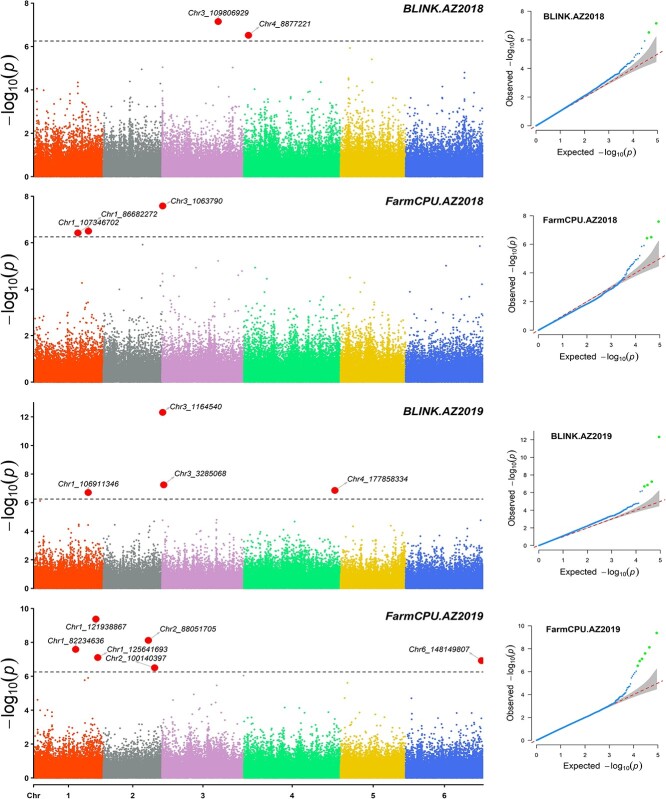
GWAS of resistance to downy mildew evaluated in the field condition in Yuma, AZ, in 2018 (AZ2018) and 2019 (AZ2019). The Manhattan and QQ plots are presented for each of the four environment trials.

Of the 434 accessions, 416 were assigned to the Q1 cluster and 18 were grouped into the Q2 cluster (Supplementary Table 1, Fig. 1a,b). The accessions from Asian countries, mainly China, and a single accession from Nepal, South Korea, Afghanistan, Thailand, and Türkiye were grouped into the Q2 sub-population. All other accessions from the remaining countries, including commercial cultivars, were grouped into the Q1 sub-population. The first two PCs used in association analysis possibly control the false positives and negatives, as shown in the QQ plots (Fig. 5, 6). The pairwise LD correlation coefficient dropped to half its maximum r^2^ at about 13.4 Kb (r^2^ = 0.391) (Fig. 1c), which is longer than previous reports in spinach [9, 54], possibly extended because of the use of a thinned set of SNPs plus GWAS associated SNPs.

### Association analysis and candidate gene search

The first step GWAS was performed with 2.91 million SNPs in TASSEL and identified 12 098 significant SNPs with LOD value >4 across different TASSEL models based on downy mildew disease scores from all four experiments (Supplementary Table 2 reports the number of SNP retained and the selection threshold from each environment). GWAS analysis was performed for the second time using the 88 682 SNP (significant SNPs identified from the first GWAS plus the 10 Kb thinned SNP) by implementing FarmCPU and BLINK models in GAPIT3. If it had a LOD value >6.25 in one of the two models, the SNP marker was selected and reported as a significant marker associated with resistance to downy mildew pathogen in this study (Table 2, Fig. 5,6).

**Table 2:** List of significantly associated SNP markers with downy mildew field resistance in spinach evaluated at multiple environments. Spinach germplasm and cultivars were screened for resistance in Salinas Valley, CA and Yuma, AZ, for two years. Genome wide association analysis (GWAS) was performed using 88 682 whole genome resequencing (WGR) generated SNP markers

SNP^a^	Chr	Position	Alleles^b^	LOD (-log_10_*P*) value in GAPIT 3^c^	GLM R^2^	MAF (%)		
BLINK	FarmCPU	GLM						
**Environment: Salinas, CA (CA2017)**								
Chr1_92 542 469	1	92 542 469	C/**T**	1.82	8.46	6.18	6.61	0.18
Chr1_103 791 151	1	103 791 151	T/**C**	9.41	5.69	6.85	7.43	0.22
Chr3_943 549	3	943 549	T/**C**	9.62	3.14	5.29	5.52	0.28
Chr3_7 984 023	3	7 984 023	C/**T**	8.07	2.02	5.67	5.99	0.20
Chr3_49 033 516	3	49 033 516	**C**/A	9.93	7.56	7.78	8.60	0.17
Chr3_101 998 183	3	101 998 183	G/**A**	7.67	2.51	3.13	2.99	0.24
Chr3_106 700 307	3	106 700 307	A/**C**	7.93	3.02	6.10	6.51	0.08
Chr3_140 181 355	3	140 181 355	C/**T**	8.52	5.08	2.85	2.66	0.12
Chr4_12 237 589	4	12 237 589	C/**T**	7.82	5.53	5.03	5.21	0.20
Chr4_159 307 591	4	159 307 591	**C**/T	11.43	6.12	5.36	5.61	0.12
Chr4_163 409 535	4	163 409 535	**T**/C	8.61	5.29	3.57	3.49	0.10
Chr5_87 443 309	5	87 443 309	**T**/A	9.06	5.44	6.14	6.55	0.04
Chr5_107 554 240	5	107 554 240	**C**/A	8.33	5.8	6.29	6.74	0.16
Chr6_35 831 188	6	35 831 188	G/**A**	6.46	4.05	1.29	0.98	0.11
**Environment: San Juan Bautista, CA (CA2018SJB)**								
Chr1_32 239 064	1	32 239 064	**C**/T	7.23	3.87	3.96	3.93	0.23
Chr1_73 968 900	1	73 968 900	**C**/A	4.24	6.49	8.23	9.13	0.32
Chr1_108 831 990	1	108 831 990	**A**/G	2.22	7.74	0.12	0.02	0.09
Chr1_112 325 794	1	112 325 794	A/**G**	1.49	7.48	3.79	3.73	0.27
Chr2_70 960 433	2	70 960 433	A/**C**	2.84	7.52	0.71	0.43	0.39
Chr2_84 580 144	2	84 580 144	A/**C**	7.64	5.31	7.73	8.49	0.22
Chr3_59 392 336	3	59 392 336	A/**C**	7.42	3.44	7.77	8.55	0.11
Chr3_104 419 739	3	104 419 739	T/**C**	9.23	8.27	9.03	10.15	0.40
Chr5_124 560 418	5	124 560 418	A/**T**	8.84	6.61	2.63	2.41	0.47
Chr6_6 118 754	6	6 118 754	G/**A**	0.35	7.60	7.49	8.20	0.33
Chr6_37 888 253	6	37 888 253	**C**/T	7.27	3.77	2.24	1.98	0.23
Chr6_96 529 106	6	96 529 106	**C**/A	3.41	7.59	2.59	2.36	0.34
**Environment: Yuma, AZ (AZ2018)**								
Chr1_86 682 272	1	86 682 272	A/**G**	4.16	6.42	5.00	5.09	0.36
Chr1_107 346 702	1	107 346 702	**C**/T	3.00	6.50	3.07	2.87	0.07
Chr3_1 063 790	3	1 063 790	C/**T**	5.04	7.58	7.81	8.48	0.06
Chr3_109 806 929	3	109 806 929	A/**G**	7.15	5.21	7.43	8.01	0.29
Chr4_8 877 221	4	8 877 221	A/**G**	6.52	3.02	6.89	7.34	0.10
**Environment: Yuma, AZ (AZ2019)**								
Chr1_82 234 636	1	82 234 636	**T**/C	3.37	7.59	8.33	8.57	0.33
Chr1_106 911 346	1	106 911 346	T/**C**	6.71	1.20	8.57	8.85	0.05
Chr1_121 938 867	1	121 938 867	T/**C**	0.78	9.38	4.65	4.39	0.11
Chr1_125 641 693	1	125 641 693	G/**A**	0.91	7.11	5.47	5.29	0.05
Chr2_88 051 705	2	88 051 705	**T**/G	1.82	8.12	6.50	6.45	0.25
Chr2_100 140 397	2	100 140 397	**A**/G	1.79	6.50	2.15	1.75	0.27
Chr3_1 164 540	3	1 164 540	G/**T**	12.31	1.99	13.15	14.40	0.30
Chr3_3 285 068	3	3 285 068	**A**/T	7.24	3.44	3.55	3.20	0.10
Chr4_177 858 334	4	177 858 334	T/**C**	6.86	3.48	12.96	14.16	0.32
Chr6_148 149 807	6	148 149 807	C/**T**	1.04	6.92	9.35	9.77	0.05

a SNP name defined as SNP position on the chromosome.

b Favorable alleles are in bold font.

c LOD (-LOG_10_*P*) value, with *P* value from the BLINK, FarmCPU, and GLM models in GAPIT 3 R package.

The quantile-quantile (QQ) plot distribution of the observed vs. expected LOD values showed a large deviation from the expected line implying associations of the SNP marker with resistance to downy mildew pathogen (Fig. 5,6). A total of 14, 12, 5, and 10 SNPs were significantly associated with resistance to downy mildew pathogen from CA2017, CA2018SJB, AZ2018, and AZ2019 experiments, respectively (Table 2). The associated SNP showed R-square values ranging from 0.02 to 14.40 with an average of 5.52% (Table 2). Several hundred SNPs were associated with other models, including the GLM model in GAPIT (data not shown), but the LOD values and R^2^ values are reported only for SNPs association identified by the BLINK and FarmCPU models.

SNP markers associated with the field resistance from four GWAS panels were distributed on all six spinach chromosomes (Table 2). From the CA2017 experiment, SNP located on chromosomes 1, 3, 4, 5, and 6 were associated with the resistance. SNPs from chromosomes 1, 2, 3, 5, and 6 were associated with resistance from the CA2018SJB trial. Similarly, from the AZ2018 trial, SNP located on chromosomes 1, 3, and 4 were associated with the resistance. And for the AZ2019 trial, SNP from chromosomes 1, 2, 3, 4, and 6 were associated. Two SNP markers (Chr3_1 164 540 and Chr 4_159 307 591) showed strong resistance with LOD > 10, indicating major resistance. Of the two SNP markers, Chr3_1 164 540 showed LOD > 11 on BLINK and > 6 on the FarmCPU models from CA2017 and Chr 4_159 307 591 showed LOD > 12 in BLINK and LOD =1.99 in FarmCPU models from AZ2019, and the former was stable in both BLINK and FarmCPU models while the latter was not stable in FarmCPU models.

SNP markers Chr3_943 549, Chr3_1 063 790, and Chr3_116450 associated in CA2017, AZ2018, and AZ2019 environments lying between 0.94 to 1.6 Mb of chromosome 3 were within the major DM R gene regions as reported in previous studies [7, 8, 10–12]. The SNP Chr4_177 858 334 identified here was 2.53 Mb away from one of the peak SNP associated with resistance to downy mildew pathogen in the report of Cai et al. (2021) [20]. None of the associated SNP markers overlapped between environments in this study, although some associated in one environment were close to the SNP associated in other environments. The SNP Chr1_103 791 151 associated in CA2017 is physically 5.04 Mb away from Chr1_108 831 990 associated in CA2018SJB. Chr3_106 700 307 associated in CA2017 is 2.28 Mb away from Chr3_104 419 739 associated in CA2018SJB. Chr6_35 831 188 associated in CA2017 is 2.05 Mb away from Chr6_37 888 253 associated in CA2018SJB. Chr1_86 682 272 associated in AZ2018 is 4.4 Mb away from Chr1_82 234 636 associated in AZ2019. Chr1_107 346 702 associated in AZ2018 is 0.43 Mb away from SNP Chr1_106 911 346 associated in AZ2019. Although in a large range of 0.43–5.04 Mb between associated SNP markers, these regions appear stably providing resistance against downy mildew pathogen. The SNPs Chr1_121 938 867 and Chr1_125 641 693 associated with AZ2019 are physically 3.70 Mb apart, SNPs Chr3_101 998 183 and Chr3_106 700 307 associated with CA2017 were only 4.70 Mb apart, and two SNP Chr1_108 831 990 and Chr1_112 325 794 associated in CA2018SJB were 3.49 Mb apart, making these regions promising in regulating field resistance against downy mildew pathogen. Some of the associated SNP markers showed relatively large differences in LOD values among the tested association models, and they were not stable across the tested models, but many that showed consistently higher LOD will be of value in molecular breeding and are practical for marker-assisted selection.

Many associated SNPs reported here contain genes within a 50 Kb distance (Supplementary Table 3). Fourteen genes were less than 2.5 Kb away from the associated SNPs, and eight overlapped over the seven associated SNPs. Chr3_943 549 associated with CA2017 is 11.3 Kb from SOV3g000980 (NB-ARC disease resistance protein) and Chr3_1 063 790 associated in AZ2018 is 7.5 Kb from SOV3g001030 (NB-ARC disease resistance protein). These two genes (SOV3g000980 and SOV3g001030) encode NBS-LRR proteins and are considered potential candidate genes to regulate resistance to downy mildew pathogen in spinach [8, 10–12, 20]. In addition, another SNP Chr3_3 285 068 associated with AZ2019 is 25.9 Kb from SOV3g003310 (putative disease resistance protein) and 37.2 Kb from SOV3g003330 (Protein kinase family protein), and the contribution of this SNP region to resistance to downy mildew pathogen is reported for the first time in spinach.

### Genomic prediction assessment

**Figure 7:**
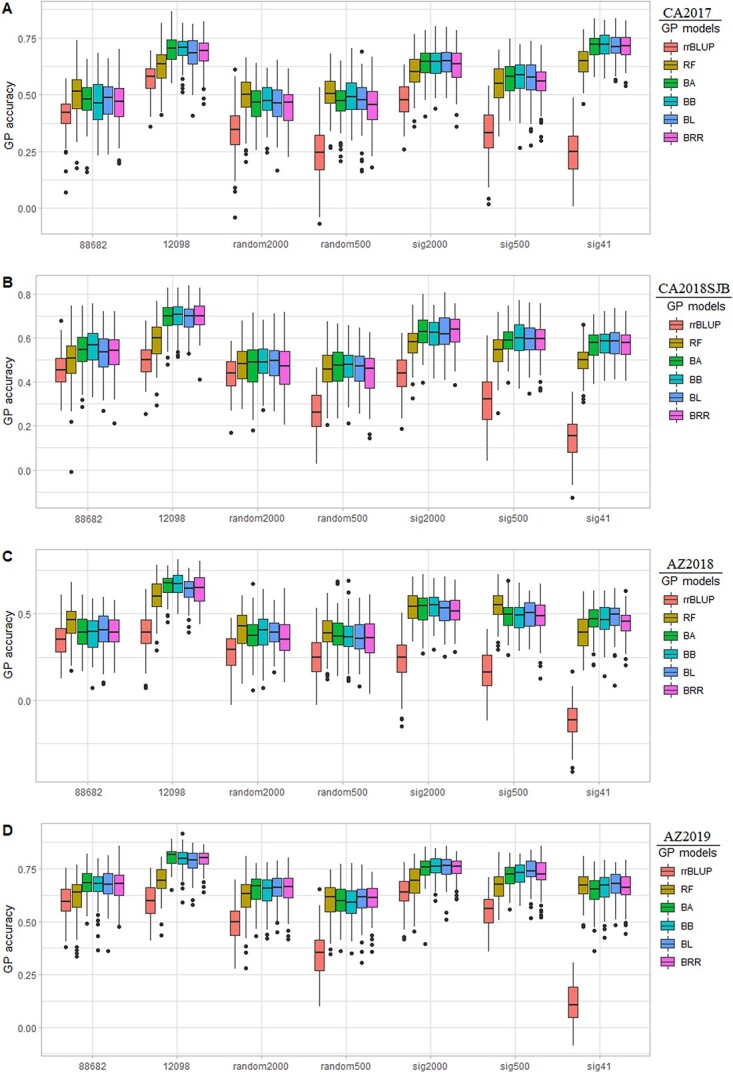
Prediction accuracy estimates following five-fold cross-validation implemented in six GP models for resistance to downy mildew evaluated in field conditions in Salinas, CA (CA2017), San Juan Bautista (CA2018SJB), and Yuma, AZ in 2018 (AZ2018) and 2019 (AZ2019). Prediction accuracy was calculated for seven marker datasets for disease scores from each environment.

GP for resistance to downy mildew pathogen was performed for all four environments with five-fold cross-validation using six GS models and seven different marker sets: 88682 SNP datasets and a random subset of 2000 and 500 markers, 12 K GWAS associated SNPs set, a random subset of 2000 and 500 markers, and 41 GWAS associated SNPs, showed wide variations in PA among models across thee environments and marker sets (Table 3, Fig. 7).

**Table 3:** Genomic prediction (r-value) evaluated with six GP models for downy mildew resistance using different marker sets across four environments

GP Model		rrBLUP	RF	BA	BB	BL	BRR	Average (All GP model)	Average (Bayesian model)
**Environment: Salinas, CA (CA2017)**									
88 682	mean	0.41	0.50	0.48	0.46	0.48	0.47	0.47	0.47
	SE	0.009	0.010	0.009	0.010	0.008	0.010		
12 098	mean	0.57	0.63	0.70	0.70	0.68	0.68	0.66	0.69
	SE	0.007	0.008	0.006	0.006	0.008	0.007		
random2000	mean	0.34	0.49	0.46	0.48	0.46	0.45	0.45	0.46
	SE	0.011	0.009	0.008	0.008	0.009	0.008		
random500	mean	0.25	0.50	0.47	0.49	0.47	0.45	0.44	0.47
	SE	0.011	0.008	0.009	0.008	0.010	0.010		
sigs2000	mean	0.48	0.60	0.64	0.64	0.64	0.63	0.61	0.64
	SE	0.008	0.008	0.007	0.007	0.006	0.008		
sigs500	mean	0.33	0.54	0.57	0.58	0.57	0.55	0.52	0.57
	SE	0.011	0.009	0.008	0.008	0.009	0.008		
sigs41	mean	0.24	0.64	0.71	0.72	0.71	0.71	0.62	0.71
	SE	0.01	0.007	0.005	0.005	0.006	0.006		
Average (SNP set)		0.33	0.49	0.50	0.51	0.50	0.49	0.47	
**Environment: San Juan Bautista, CA (CA2018)**									
88 682	mean	0.45	0.49	0.55	0.56	0.53	0.53	0.52	0.54
	SE	0.008	0.011	0.009	0.009	0.01	0.009		
12 098	mean	0.50	0.59	0.69	0.70	0.69	0.69	0.64	0.69
	SE	0.007	0.009	0.007	0.007	0.006	0.006		
random2000	mean	0.43	0.48	0.48	0.49	0.48	0.47	0.47	0.48
	SE	0.008	0.009	0.011	0.009	0.009	0.01		
random500	mean	0.26	0.45	0.47	0.47	0.46	0.44	0.43	0.46
	SE	0.01	0.01	0.009	0.008	0.009	0.01		
sigs2000	mean	0.44	0.58	0.63	0.62	0.62	0.63	0.59	0.63
	SE	0.009	0.008	0.008	0.007	0.008	0.008		
sigs500	mean	0.31	0.54	0.59	0.60	0.60	0.59	0.54	0.60
	SE	0.012	0.008	0.007	0.008	0.007	0.008		
sigs41	mean	0.14	0.50	0.57	0.58	0.58	0.57	0.49	0.58
	SE	0.009	0.007	0.007	0.007	0.007	0.006		
Average (SNP set)		0.36	0.52	0.57	0.57	0.57	0.56	0.53	
**Environment: Yuma, AZ (AZ2018)**									
88 682	mean	0.35	0.45	0.40	0.39	0.40	0.40	0.40	0.40
	SE	0.011	0.011	0.01	0.01	0.01	0.009		
12 098	mean	0.39	0.60	0.66	0.67	0.64	0.64	0.60	0.65
	SE	0.011	0.01	0.007	0.007	0.007	0.008		
random2000	mean	0.27	0.41	0.38	0.40	0.40	0.36	0.37	0.39
	SE	0.011	0.011	0.01	0.011	0.008	0.011		
random500	mean	0.25	0.39	0.38	0.37	0.37	0.36	0.35	0.37
	SE	0.012	0.01	0.01	0.011	0.01	0.011		
sigs2000	mean	0.23	0.54	0.53	0.54	0.52	0.52	0.48	0.53
	SE	0.013	0.009	0.009	0.008	0.009	0.009		
sigs500	mean	0.17	0.54	0.49	0.48	0.50	0.48	0.44	0.49
	SE	0.013	0.009	0.008	0.008	0.008	0.01		
sigs41	mean	−0.12	0.39	0.46	0.46	0.48	0.45	0.35	0.46
	SE	0.01	0.011	0.009	0.01	0.009	0.009		
Average (SNP set)		0.22	0.47	0.47	0.47	0.47	0.46	0.43	
**Environment: Yuma, AZ (AZ2019)**									
88 682	mean	0.60	0.61	0.68	0.67	0.67	0.67	0.65	0.67
	SE	0.008	0.01	0.007	0.007	0.008	0.007		
12 098	mean	0.60	0.70	0.80	0.79	0.79	0.79	0.75	0.79
	SE	0.008	0.007	0.004	0.005	0.005	0.004		
random2000	mean	0.49	0.62	0.66	0.64	0.66	0.66	0.62	0.66
	SE	0.009	0.01	0.007	0.007	0.006	0.008		
random500	mean	0.35	0.60	0.60	0.59	0.60	0.61	0.56	0.60
	SE	0.01	0.009	0.008	0.008	0.008	0.007		
sigs2000	mean	0.64	0.68	0.75	0.76	0.76	0.75	0.72	0.76
	SE	0.007	0.008	0.006	0.005	0.006	0.005		
sigs500	mean	0.55	0.67	0.72	0.72	0.74	0.72	0.69	0.73
	SE	0.008	0.007	0.006	0.006	0.006	0.007		
sigs41	mean	0.11	0.67	0.64	0.66	0.68	0.66	0.57	0.66
	SE	0.009	0.007	0.008	0.008	0.006	0.007		
Average (SNP set)		0.48	0.65	0.69	0.69	0.70	0.69	0.65	

#### Prediction accuracy with different genomic selection models

Average PA from 100 runs among the tested models for the 88 682 SNP set ranged from 0.41–0.48 for CA2017, 0.45–0.56 for CA2018SJB, 0.35–0.45 for AZ2018, and 0.60–0.68 for AZ2019 (Table 3). Similarly, for the 88 682 datasets, AZ2019 provided the highest PA of 0.65, followed by CA2018SJB, CA2017, and AZ2018 with PA of 0.52, 0.47, and 0.40.

PA from the rrBLUP model ranging from 0.35 to 0.60 was consistently lower in all four environments for 88 682 SNP datasets than in other GS models (Table 3, Fig. 7). The random forest model showed PA in the range of 0.45 to 0.61 for 88 682 SNP datasets across four environments, which is slightly lower or comparable to other Bayesian models. The PA from four Bayesian models, BA, BB, BL, and BRR, was similar but higher than other models, with the PA ranging between 0.39 to 0.68 for the 88 682 SNP datasets. On the 88 682 SNP dataset and all other SNP datasets, PA was higher for Bayesian models than the rrBLUP model. Thus, these Bayesian models appear more appropriate for conducting GS for resistance to downy mildew pathogen in spinach.

Comparing the average PA of the seven datasets for different environments, rrBLUP averaged 0.33, 0.36, 0.22, and 0.48 for CA2018, CA2018SJB, AZ2018, and AZ2019; meanwhile, the PA from the RF model was 0.49, 0.52, 0.47, 0.65, respectively for these environments (Table 3). The rrBLUP model provided the lowest PA in all environments and marker sets. The PA of four Bayesian models averaged 0.50, 0.57, 0.47, and 0.69 across all four environments. In general, PA of the Bayesian models was higher among the tested models in all environments and across all marker sets and appeared to predict the resistance to downy mildew pathogen with higher efficiency, although RF models provided comparable PA. Interestingly, the PA obtained using RF models was inferior to Bayesian models in large SNP datasets (80 K and 12 098 SSNP set) but equivalent to or higher for random subsets of markers (random2000 and random500). Among the Bayesian models, the BB and BL predicted the resistance to downy mildew pathogen with higher accuracy, making them a more suitable choice to conduct GS to select for resistance.

#### Effect of different marker sets on prediction accuracy

GP models were tested using seven different marker sets to determine if a small number of markers could achieve comparable PA as the full datasets. Based on the higher predictive ability of Bayesian models over others, the results in this section are presented as the mean PA across Bayesian models only.

GP performed using full 88 682 SNP dataset resulted in PA of 0.47, 0.54, 0.40, 0.67 in CA2017, CA2018SJB, AZ2018, and AZ2019 (Table 3). The predictability of the full 88 682 datasets was lower than the significant 12 098, sig2000, and sig500 marker sets (Table 3, Fig. 7). Interestingly, GWAS-associated 12 098 marker set showed an average PA of 0.69, 0.69, 0.65, 0.79 in CA2017, CA2018SJB, AZ2018, and AZ2019, providing consistently higher PA in all locations except CA2017, where the 41 SNP sets exceeded all other marker sets (Table 3, Fig. 7). PA from random2000 was higher than random500 in all evaluated environments. But, both the random marker subsets yielded lower PA than the sig2000, sig500, and 88 682 SNP sets in all environments. The PA of sig2000 marker set ranked second in CA2018SJB (r = 0.63), AZ2018 (r = 0.53), and AZ2019 (r = 0.76) and ranked third in CA2017 (r = 0.64). Similarly, the PA of the sig500 marker set was 0.57, 0.60, 0.49, and 0.73 for CA2017, CA2018SJB, AZ2018, and AZ2019 environments, respectively. Sig41 was higher or equivalent to random500 and random2000 marker sets based on Bayesian models across all environments. Further, the sig41 marker set (r = 0.71) outperformed all other marker sets in CA2017, and it is still reasonable to use this marker set in GS based on PA obtained in other environments (Table 3, Fig. 7).

Excluding the 88 682 SNP sets, decreasing the number of markers reduced PA in both subsets of random and GWAS-associated markers. A random marker set without incorporating GWAS-associated SNPs resulted in lower PA, while the inclusion of GWAS-associated SNP increased prediction power, even when just 41 GWAS SNP was used. Overall, PA of sig2000, a subset of GWAS markers, remained high and is the best marker set; thus recommended to use this set, as it provides adequate PA for all locations, compared to a large number of 12 098 SNP set. Nevertheless, the PA obtained from sig500 and sig41 is good enough to predict resistance to downy mildew pathogen with a slight reduction in prediction potential (0.64 and 0.57 vs. 0.71 in CA2017, 0.63 and 0.60 vs. 0.58 for CA2018SJB, 0.53 and 0.49 vs. 0.46 for AZ2018, 0.76 and 0.73 vs. 0.66 for AZ2019), and these small SNP sets appear to be cost-effective compared to genotyping a more extensive set of 2000 or more markers (Fig. 7).

## Discussion

### Downy mildew field resistance in spinach germplasm

Downy mildew has been the most devastating disease of spinach worldwide, particularly in the Salinas Valley in California and the arid regions of California and Arizona in the United States, representing over 90% of the US spinach production. Due to the high-quality standards of spinach, less than 2% of the crop with downy mildew symptoms is typically tolerated. A number of RPF alleles have been identified that have a major gene effect on various races of the spinach downy mildew pathogen (Correll et al., 2011). However, new races are continually overcoming the genetic resistance deployed in the newer cultivars (Correll et al., 2011; Feng et al., 2018b; Bhattarai et al., 2020). Disease resistance to the downy mildew pathogen is even more critical for organic spinach production as it represents the only effective disease management tool. Organic spinach production represents approximately 50% of the total spinach production in the United States, thus necessitating disease-resistant cultivars. The ability of the pathogen to overcome the major resistance genes in spinach has provided the impetus for the characterization of quantitative resistance and identifying QTLs with minor effects to further manage downy mildew disease by combining both qualitative and quantitative host genetic resistance.

Towards achieving the aim of utilizing genetic resistance in downy mildew disease management, the spinach germplasm panel was evaluated for resistance to the downy mildew pathogen under field conditions with natural disease pressure in five environments over two years and in two major spinach producing regions in the US: Salinas, California and Yuma, Arizona. A wide range of continuous downy mildew disease responses was observed among the accessions in all experimental trials, reflecting the substantial genetic variation in the evaluated panel. In addition, the disease response was highly variable across environments, with the Pearson correlation coefficient ranging between 0.03–0.52 between environments. During the field experiments, it is likely that the pathogen population was composed of multiple races and novel strains of the downy mildew pathogen in the various locations [3, 55]. A similar large variation in disease severity was observed among the commercial cultivars that were screened in the same locations in different trials [51–53]. Contrarily, field resistance evaluation of lettuce germplasm panel against downy mildew pathogen *Bremia lactucae* reported relatively stable disease response across locations and years and identified QTLs associated with the resistance via GWAS approach [21]. This study aimed to identify germplasm with higher levels of resistance to the downy mildew pathogen across all tested environments. However, only a few accessions (PI 433210, Califlay, PI 648950, PI 173130, PI 165560, PI 677109, Whale, PI 169679, PI 173809, NSL 81328, NSL 32678, Boeing, PI 166366, PI 274046, NSL 184379, and PI 531456) showed relatively stable high resistance across tested environments (Fig. 4). In contrast many others showed variable response between environments. Indeed, the lines that appeared to be more stable in the reactions in this study could be used to introgress beneficial alleles to improve resistance against downy mildew pathogen. However, some of the lines tested contain known RPF genes effective against particular races of *P. effusa*. Further, the intermediate to high heritability estimates obtained in this study for each environment suggests that a high portion of the downy mildew disease variation among the genotypes was genetically controlled and indicates potential breeding prospects and genetic improvement following breeding and selection.

The GWAS panel was evaluated across a highly heterogeneous environment for downy mildew disease pressure and pathogen races, which resulted in a variable disease rating of spinach accessions across environments, and led to high GxE and low to moderate heritability. Such differential disease reactions and high GxE interactions are common for disease resistance traits because the predominance of pathogen populations continually varies across environments, as discussed by others [19]. Different environmental conditions fluctuate downy mildew pathogen pressure, and the predominance of pathogen races widely varies at each location, leading to a wide variation in disease response across years and locations [3, 4, 6, 56]. Thus, the downy mildew disease response data across environments were independently used for GWAS and GS and tested with multiple association models to increase the confidence of associated SNPs.

### Genomic regions controlling downy mildew field resistance

A set of 41 SNPs, distributed on all six spinach chromosomes, were associated with the field resistance to the downy mildew pathogen from four environments (Table 2). Some SNPs (Chr3_943 549 and Chr3_1 063 790), associated with the field resistance, are located near the known RPF loci in the proximal end of chromosome 3, consistent with previous mapping studies [8, 10–12, 20] that have localized the resistance region via QTL and association mapping approaches. Most of the other associated SNP markers identified in this study were located in the chromosome regions not previously reported, and many associated SNPs were less than 5 Mb apart within/across environments. These novel SNPs and QTLs associated with resistance to the downy mildew pathogen may be valuable for future breeding efforts. The medium -log_10_(P) and R square values of the SNPs associated with the resistance to the downy mildew pathogen under natural inoculum pressure might be due to a small number of markers used in this study, or the associated SNPs were further apart from the candidate loci. There is a potential to pyramid effective QTLs identified from field-evaluated spinach germplasm with major RPF genes to provide a more broad-spectrum and durable resistance against the downy mildew pathogen [57–59].

The resistance QTL and associated SNP markers are suitable for MAS as direct selection markers or flanking markers to develop more closely linked markers. Meanwhile, the identified QTL are proposed for validation in additional populations and environments. However, the disease responses across locations lack stability, so the associated QTLs and some of the associated SNP markers were inconsistent across the tested models. The GWAS analysis from multiple environments suggests a complex genetic basis of field resistance at each tested environment and extensive GxE interactions. This lack of detecting stable QTLs across environments may be due to the influence of a genetically diverse pathogen population, level of disease pressure, and environment over years and locations [19, 60–62]. Indeed, quantitative disease resistance is often challenging to evaluate for disease incidence and severity under field conditions [19, 63]*.* Thus, identifying linked loci and subsequent gene introgression in the breeding lines may remain an ambitious plan when both qualitative and quantitative genes are the target of selection.

### Assessment of genomic prediction accuracy

GS has recently been evaluated in spinach for white rust resistance [36] and some other phenotypes are being assessed for GS in spinach [56], including this downy mildew field resistance study. Implementing GS will be a practical and attractive option depending on low-cost genotyping resources and highly accurate multi-environment phenotype datasets that provide increased PA. The GS prediction models have different assumptions to treat marker effects, so the PA differs based on the phenotype and genetic architecture of the trait. GP was explored to determine PA for resistance to downy mildew using six GP models involving parametric models (rrBLUP, BA, BB, BL, and BRR) and non-parametric models (RF). Overall, Bayesian models showed consistently higher PA followed by the RF model. But the rrBLUP showed low PA in all tested environments, possibly because of a lack of strong population structure and large effect QTLs in spinach panels [35, 64, 65]. The PA corresponded to the heritability estimates for each environment, indicating the worth of additive genetic variance in estimating GEBV, as reported in previous studies [31, 39, 65, 66]. Bayesian models provide higher PA for traits controlled by a few major QTLs with large effects [64], while the rrBLUP considers equal variances of all markers and incorporates genetic relationships, and low PA was reported for some traits in previous studies [35, 67]. The higher PA of Bayesian models and lower PA of rrBLUP for downy mildew field resistance appears to be because of large-effect QTLs associated with resistance in this study, as reported in the GWAS section. The GWAS panel comprised 7 to 9 differentials cultivars in all environments and around 35 commercial cultivars in AZ2018 and AZ2019. These cultivars contain the RPF alleles and are resistant to some of the races of the downy mildew pathogen, which have led to SNP associations in the 0.9–1.2 Mb region of Chromosome 3 (in CA2017, AZ2018, AZ2019) known to harbor the RPF genes. Thus, high PA was obtained for Bayesian models because of the presence of major QTLs in the training set of this study.

Additionally, PA was investigated using seven different sets of markers to identify small marker subsets that can effectively predict the breeding value. PA of the 88 682 SNP set was lower than the 12 098 set, which may be due to the overfitting of the GS model for the large number of SNPs in the 88 682 dataset. A recent study reported a similar conclusion when both major and minor QTLs were incorporated for stripe rust resistance in wheat [63]. PA for a more extensive marker set was higher for both the random and significant marker subsets, but still, the increment was minimal between marker numbers of 2000 and 500 (difference of 0.02 between random2000 vs. random500 and of 0.04 between sig 2000 vs. sig 500). The GWAS associated SNP set showed improved PA for all locations. At the same time, a relatively small number of GWAS markers estimated comparable prediction as that of the full set as the use of 2000 GWAS SNP achieved similar PA as using 12 K SNP (0.69 vs. 0.64 for CA2017, 0.69 vs. 0.63 for CA2018SJB, 0.65 vs. 0.53 for AZ2018, and 0.79 vs. 0.76 for AZ2019), as this allows using a small marker set with minimal effect on predictive ability. Such comparable PA obtained from a small SNP panel is attractive for adoption as it minimizes the cost of genotyping and favors the use of a small number of GWAS associated SNP in GS. Overall, smaller sets of markers associated with the QTLs show improved PA in all GS models; thus, identifying effective QTLs associated markers and using them will facilitate the practical implementation of GS in breeding programs. We did not test and evaluate the optimum number of the training population sizes in this study, but another GS study on white rust resistance in spinach found that changing the training and testing size from 2 to 10 fold does not make much difference in PA [36].

This study aimed to identify field resistance to the downy mildew pathogen under a natural disease pressure, identify and map the partial resistance governed by the putative QTL, and evaluate prediction accuracy performance using multiple GP models. Quantitative resistance, controlled by multiple genes with a small effect, is often affected by environmental factors (temperatures, moisture, pathogen populations) and hence is challenging for a breeder to utilize GP for such genes or loci [19]. GS is continually being optimized and appears to be a more effective selection method for quantitative traits governed by small to large effect loci and possibly in the presence of major loci [19, 63]. Field resistance to the downy mildew of spinach is genetically complex, regulated by some major loci and several minor loci in providing resistance. This study finds the prediction accuracy in a range of 0.65–0.79 using 12 098 SNP markers (selected from GWAS step 1) and 0.53–0.76 with sig2000 SNP markers (selected 2000 GWAS markers) in the four environments, demonstrating a moderate to higher prediction accuracy for the resistance to downy mildew with a relatively small number of marker sets. GP evaluated for many disease resistance traits in other crops reported PA in the range of 0.4 to 0.8 [62, 68–70]. Despite the challenges, a systematic approach of screening for new resistance genes and deployment of the most promising R genes and QTLs in a combination, using GP models as reported in this study, may allow the development of potentially more durable resistant cultivars.

## Conclusions

The resistance to the spinach downy mildew pathogen was evaluated under field conditions across multiple environments. The GWAS analysis indicates the presence of several moderate effects QTL, which can provide moderate levels of resistance. We identified 14, 12, 5, and 10 SNP markers (LOD value above 6.25) significantly associated with resistance to the downy mildew pathogen in one of the tested GWAS models from CA2017, CA2018SJB, AZ2018, and AZ2019 trials, respectively. Some of the associated SNPs were within the known genomic regions where major genes for resistance have been located on the proximal end of chromosome 3, particularly 7.5 and 11.3 Kb from SOV3g001030 and SOV3g000980 encoding NBS-LRR proteins [20]. Bayesian models performed better in predicting GEBV across all environments. Prediction accuracy from the full 88 682 SNP set (average PA of the Bayesian models across all environments was 0.52) was lower than other datasets. The use of GWAS-associated small subset of SNPs comprising sigs2000, sigs500, and sigs41 showed PA of 0.64, 0.59, and 0.60, which was lower but comparable to the highest predicting set of 12 098 SNPs with PA of 0.71, providing a more attractive option of using small sets of markers in GP to improve spinach for resistance to the downy mildew pathogen. The identification and utilization of quantitative resistance in spinach may help reduce disease pressure and may help delay the time it takes for the pathogen to overcome the deployed major genes for resistance. Evaluation and quantification of the genetic variation of germplasm collections for resistance to the downy mildew disease pathogen and further identification and validation of molecular markers may enhance the efficiency of developing spinach cultivars with improved durable resistance. Understanding spinach-downy mildew pathogen interaction, the virulence evolution of the downy mildew pathogen, and the functional characterization of genetic resistance are some of our targeted approaches to advancing molecular-genomic resources toward implementing genetic resistance. This study generated new information and molecular resources to breed spinach with improved resistance to the downy mildew pathogen.

## Materials and methods

### Plant material

The spinach association panel used in this study comprised 434 spinach genotypes, including 381 USDA accessions and 53 commercial cultivars. The USDA accessions were obtained from the germplasm repository at the North Central Regional Plant Introduction Station (NCRPIS), USDA-ARS, Ames, IA. The spinach germplasm used in this study was initially collected from 34 countries with ten or more accessions from Turkey, the US, Afghanistan, Macedonia, Iran, China, India, Belgium, and Syria (Supplementary Table 1). The worldwide distribution of these germplasm panels presents a wider phenotypic variation on economically important traits, as documented for several phenotypes in the USDA GRIN database. Around ten seeds per genotype were sown in pots in the greenhouse at the University of Arkansas and bulked for DNA extraction and sequencing.

### Downy mildew field trails and phenotype evaluation

The diverse spinach panel was screened for downy mildew disease severity in the field condition under natural inoculum pressure for two years at the USDA research station in Salinas, CA, defined the two experiments as CA2017 and CA2018, two years at the Yuma agricultural center, the University of Arizona, AZ, defined as AZ2018 and AZ2019, and one year at the Seminis vegetable seeds research station in San Juan Bautista, CA, defined as CA2018SJB. Field evaluation trials were conducted from September–October in CA and January–March in AZ. These experimental areas are the major commercial spinach production regions in the US and present high pathogen pressure and favorable environmental conditions for disease development every year. Spinach genotypes were planted in a single row with 15–30 seeds in a randomized complete block design with two replications for each experimental trial. Each plant row was 1.5 m in length and 0.3 m between rows. Plants were watered 2–3 times a week with overhead sprinklers during the experimental trial. The cultivar Viroflay, susceptible to all known races of *P. effusa,* was planted in the border rows vertical to the test rows.

Individual plants for each genotype were visually scored for the presence of signs and symptoms of downy mildew between 35–45 days from planting. Individual plants were scored for disease severity (DS) on a 0–100% scale, representing the total percentage of infected leaf area, with 0% meaning no symptom and 100% representing complete infection in all environments except in AZ2018. In AZ2018, disease incidence (DI) was scored by row (recording the number of infected vs. clean plants). We did not use disease response from the CA2018 trial because of the low disease severity rating across the evaluated panel.

### Phenotype data analysis

Disease scores from each experimental trial were analyzed independently to account for large genotype-by-year interactions as the predominance of races and pathogen pressures vary among years and locations. The random effect model was fitted to the mean disease score by considering genotype and block as random factors using the lme4 package implemented in META-R v6.0.4 (Alvarado et al., 2020). The BLUPs values were obtained as

$$ {\textrm{Y}}_{ij}=\upmu +{\textrm{Rep}}_i+{\textrm{Gen}}_j+{\varepsilon}_{ij} $$

where Y*ij* is the phenotype, μ is the mean effect, Rep*i* is the effect of replicate *i*, Gen*j* is the effect of genotype *j* and *Ɛij* is the residual error. The BLUPs values from this model for each experiment trial were used as the phenotype dataset in association analysis. Broad-sense heritability on a genotype-mean basis was calculated using the variance component estimates from the same model, as

$$ \textrm{H}=\frac{\upsigma^2\textrm{g}}{\upsigma^2\textrm{g}+\frac{\upsigma^2e}{n\textrm{Rep}}} $$

where ${\sigma}^2$g is the genetic variance and ${\sigma}^2e$ is the prediction error variance, and *n*Rep is the number of replicates.

### Sequencing and SNP calling

Genomic DNA was extracted with Omega MagBind Plant DNA DS kit (Omega Bio-tek Inc., Norcross, GA, USA) in an automated KingFisher Flex extraction system (Thermo Fisher Scientific, Waltham, MA, USA). Extracted DNA was quantified using a Qubit Fluorometer and sample integrity was tested on 1% agarose gel electrophoresis. Paired-end sequencing libraries for 480 spinach accessions were created and sequenced on Illumina NovaSeq at Beijing Genome Institute (BGI). The whole genome resequencing (WGR) was pursued to generate around 10 Gb sequence reads per sample, approximating 10x genome coverage. Variants were called by mapping the sequence reads to Monoe-Viroflay reference genome [20] using the Illumina Dynamic Read Analysis for GENomics (DRAGEN) pipeline (v 3.8.4). SNP variants were initially filtered using BCFtools [40] for a minimum coverage depth of 6, minimum genotype quality of 10, and minor allele frequency (MAF) of 0.05. This filtering resulted in 4.92 million SNPs across 470 spinach accessions, of which six chromosomes contained 4.88 million SNPs.

Next, SNPs from six chromosomes were extracted and further filtered using BCFtools to remove monomorphic SNPs, keep only biallelic SNPs, and remove indels and SNPs within 10 bp of indels using BCFtools. Variants were filtered for more than 20% missing calls using BCFtools and genotypes above 50% missing rates using VCFtools that removed four individual lines. Variants data for 466 spinach genotypes were then filtered for heterozygosity >60% and > 10% missing calls.

Again, SNP data for 434 genotypes with phenotype data available in this study were extracted and filtered for heterozygosity >50%, retaining 2.91 million SNPs as the first GWAS variant dataset. A thinned SNPs dataset containing 76 951 SNP was extracted from the first GWAS dataset by removing SNPs within 10 Kb using VCFtools and merged with 12 098 significant SNPs identified from the first GWAS analysis (see GWAS section below). This final filtered dataset containing 88 682 unique SNPs among 434 spinach genotypes was used for genetic diversity and GWAS analysis in this study.

### Population structure and genetic diversity

PCA and genetic diversity analyses were performed using the 88 682 SNPs in GAPIT 3 [41,42] programs by setting PCA and NJ tree =2. Two clusters were chosen based on previous reports using GBS and WGR derived SNPs in similar sets of USDA spinach accessions [36, 43]. An unweighted neighbor-joining (NJ) tree was drawn in GAPIT 3. LD was computed and plotted with all SNP pairs within a 500 Kb window using PopLDdceay v3.41 [44]. Linkage disequilibrium decay was estimated as the distance the Pearson correlation coefficient (r^2^) dropped to half of the average maximum r^2^ value.

### Association analysis and candidate gene search

Initially, GWAS was performed using 2.91 million WGR generated SNPs and the phenotype scores from four environments using single marker regression (SMR), general linear model (GLM), and mixed linear model (MLM) in TASSEL 5.2.74 Linux command line [45]. Inbuilt principal components and kinship matrices were used to run SMR, GLM and MLM models in TASSEL. Significantly associated SNPs were selected with the threshold of LOD (−log_10_(*P*)) value >4 in the MLM model, 5–8 in GLM, and 6–10 in SMR models from all four environments (Supplementary Table 2), and the LOD value differences were used to adjust different numbers of SNPs showing significance across environments.

A new SNP dataset (88 682 SNP sets) was created for second stage GWAS by keeping 10 Kb thinned SNPs sets plus the unique significant SNPs identified from the first GWAS. GWAS was performed for the second time using the 88 682 SNPs using the BLINK [46], FarmCPU [47], and GLM model in GAPIT 3 [41, 42]. Significant SNPs were determined using a Bonferroni threshold of 0.05 (LOD > 6.25) for BLINK and FarmCPU models. The LOD and R^2^ values of the associated SNPs in the former two models were extracted from the GLM model and reported. The BLINK model uses iterations to select a set of markers associated with the trait in which the associated markers are fitted as a covariate for testing the remaining markers and is known to have higher statistical power than GLM, MLM and FarmCPU models [46].

Genes were searched for GWAS associated SNPs for all environments within 50 Kb on either side of the Monoe-Viroflay assembly. Predicted functions for genes in the vicinity of associated SNPs were reported, emphasizing genes predicted to provide disease resistance in plants.

### Genomic selection

GP was assessed using six different GS models and seven sets of marker datasets for resistance to downy mildew pathogen for each tested environment (CA2017, CA2018SJB, AZ2018, and AZ2019) to identify best performing models and marker sets. The GS models were ridge regression best linear unbiased prediction (rrBLUP), random forest (RF), and Bayesian models Bayes A, Bayes B, Bayesian LASSO, and Bayesian ridge regression (BRR). The rrBLUP was fitted using the rrBLUP R package [48], and RF model with 100 decision trees was run using the Random Forest R package [49], and the Bayesian models using the BGLR R package with 3000 iterations and 1500 burn-in [50].

GP was performed following a five-fold cross-validation scheme where individuals are randomly assigned into five groups, of which four groups are retained as the training set (80% of individuals), and the remaining fifth group (20% individuals) serves as the validation set to predict genomic estimated breeding values (GEBV). The cross-validations were replicated 100 times and prediction accuracy (PA) was determined by averaging the Pearson correlation coefficient (r) between predicted GEBV values obtained from five-fold cross-validations and observed phenotype values in the validation set.

GP was further assessed using seven marker sets for all six models to determine the optimum number of markers to obtain high PA for resistance to downy mildew pathogen. The first marker set was the 88 682 SNPs used for GWAS analysis, and the second set contained 12 098 SNP markers associated with resistance to downy mildew pathogen across tested environments in the first GWAS analysis. The other two sets, random2000 and random500, are subsets of random 2000 and 500 SNPs from the 88 682 SNP sets. Similarly, sig2000 and sig500 comprise random 2000 and 500 SNPs from the 12 098 SNP set. And the sig41 contains the significantly associated SNPs identified from the GWAS analysis (see GWAS result below).

## Acknowledgments

The authors would like to acknowledge funding support in part from the USDA-AMS and USDA-SCRI, all support to evaluate the germplasm panel at USDA-ARS CIPRU, Salinas, CA; Seminis vegetable seeds research station in San Juan Bautista; and Yuma agricultural center, the University of Arizona, Yuma, AZ. This research was supported by USDA-SCRI grant 2017-51181-26830, USDA-AMS SCMP grant 16SCCMAR0001, and USDA NIFA Hatch project ARK0VG2018 and ARK02440. A.S. managed funding.

## Author Contributions

A.S., B.M., J.C., G.B. conceived the study. A.S. supervised and managed funding. G.B. conceptualized methodology and data analysis. GB and all authors performed or managed evaluation experiments and phenotype data collection. G.B. performed computational analysis, analyzed the data, wrote the original draft, and reviewed and edited the manuscript. A.S., B.M., J.C. reviewed and edited the manuscript. All authors read, revised, and approved the final manuscript.

## Data availability

Data generated in this study are available in the main table, figures, and additional files. The SNP datasets are available in the Figshare repository, https://doi.org/10.6084/m9.figshare.19773070.v1 (Accession number: 19773070). Genome sequence raw data generated for all spinach accession were deposited to the NCBI Sequence Read Archive (SRA) under BioProject accession: PRJNA860974.

## Conflict of Interest

The authors declare that the research was conducted in the absence of any commercial or financial relationships that could be construed as a potential conflict of interest.

## Supplementary data

Supplementary data is available at * Horticulture Research* online.

## Supplementary Material

Web_Material_uhac205
